# Retraction: GATA4 regulates inflammation-driven pancreatic ductal adenocarcinoma progression

**DOI:** 10.3389/fcell.2023.1260259

**Published:** 2023-09-15

**Authors:** Frontiers Editorial Office

Following publication, concerns were raised regarding the integrity of the images in the published figures. The authors failed to provide a satisfactory explanation during the investigation, which was conducted in accordance with Frontiers’ policies.

As a result, the data and conclusions of the article have been deemed unreliable and the article has been retracted.

The co-authors, Dr. Weiliang Jiang and Dr. Jie Shen agreed to this retraction.

This retraction was approved by the Chief Editor of Frontiers in Cellular and Developmental Biology and the Editor-in-Chief of Frontiers.

